# Supplementary material for: Short-Term Transcriptomic Points of Departure Are Consistent with Chronic Points of Departure for Three Organophosphate Pesticides across Mouse and Fathead Minnow
Source: Toxics. 2023 Sep 29;11(10):820. doi: 10.3390/toxics11100820 (PMC10611195; doi:10.3390/toxics11100820)
Supplement: Supplementary file 1 [file toxics-11-00820-s001.zip › toxics-2617159-supplementary.pdf]

toxics

MDPI

Supplementary Materials: Short-Term Transcriptomic Points of Departure Are Consistent with Chronic Points of Departure for Three Organophosphate Pesticides across Mouse and Fathead Minnow

Rubia Martin, Monique Hazemi, Kevin Flynn, Daniel Villeneuve and Leah Wehmas

Liquid Chromatography Mass Spectrometry (LC-MS)

Samples were analyzed for chemical concentration using liquid chromatography mass spectrometry (LC-MS) using a triple quadrupole mass spectrometer with electrospray ionization. Full methodological details and method performance metrics are included in the Supporting Information.

Measurement of parathion, fenthion, and methidathion were carried out using a Thermo Scientific LC-MS system consisting of a Vanquish LC system coupled to a TSQ Altis tandem MS. To obtain separation of compounds, a 1 uL injection was separated under gradient flow on an Agilent ZORBAX Eclipse Plus C18 column (50 mm x 2.1 mm, 1.8 um) using water with 5 mM ammonium formate and 0.1% formic acid (solvent A) and methanol with 5 mM ammonium formate and 0.1% formic acid (solvent B). With a constant flow rate of 0.4 mL/min, gradient conditions began at 25% B, increased to 90% B from 0 to 3 minutes, held at 90% B from 3 to 4.1 minutes, followed by a 2 minute equilibration at 25% B. Analytes were detected using electrospray ionization (ESI) in positive polarity, and ion source parameters including spray voltage (4500 V), sheath gas (56.8 Arb), auxiliary gas (1 Arb), sweep gas (1.6 Arb), ion transfer tube temperature (325°C), and vaporizer temperature (350 °C) were optimized using parathion as a reference compound. Under these conditions, retention time, precursor mass, product ion masses (qual/quant), and collision energy for each target analyte were: parathion: 3.61 min, 292.1 m/z, 236/264 m/z, 15 V; fenthion: 4 min, 279 m/z, 169/247 m/z, 18 V; methidathion: 3.08 min, 303 m/z, 85/145 m/z, 21 V. Detection limit for this method is 1.0 ng/mL for all analytes.

Standards were prepared in 50:50 water:isopropyl alcohol (except the highest concentration standard of 500 ng/mL, which was in 25:75 water:isopropyl alcohol). Parathion certified standard in cyclohexane was purchased from Sigma-Aldrich (Saint Louis, MO) at 99.3% purity. Fenthion and methidathion certified standards in methanol were purchased from AccuStandard, Inc. (New Haven, CT) at 98.3% purity. Mass labeled parathion internal standard was ordered and used initially, but the standard curve was much more linear without it, so it was not included in future sample preparation or analysis. A standard curve was run before each set of samples, and check standards were run every 15 samples. Using the 500 ng/mL standard and spiking into 50:50 water:isopropyl alcohol, three spikes were prepared at 50 ng/mL, and three spikes were prepared at 250 ng/mL. Three method spikes were prepared by spiking 500 ng/mL standard into a sample. Spike recoveries ranged from 85-113% for parathion, 87-116% for fenthion, and 88-112% for methidathion.

SUPPLEMENTAL FIGURE LEGENDS

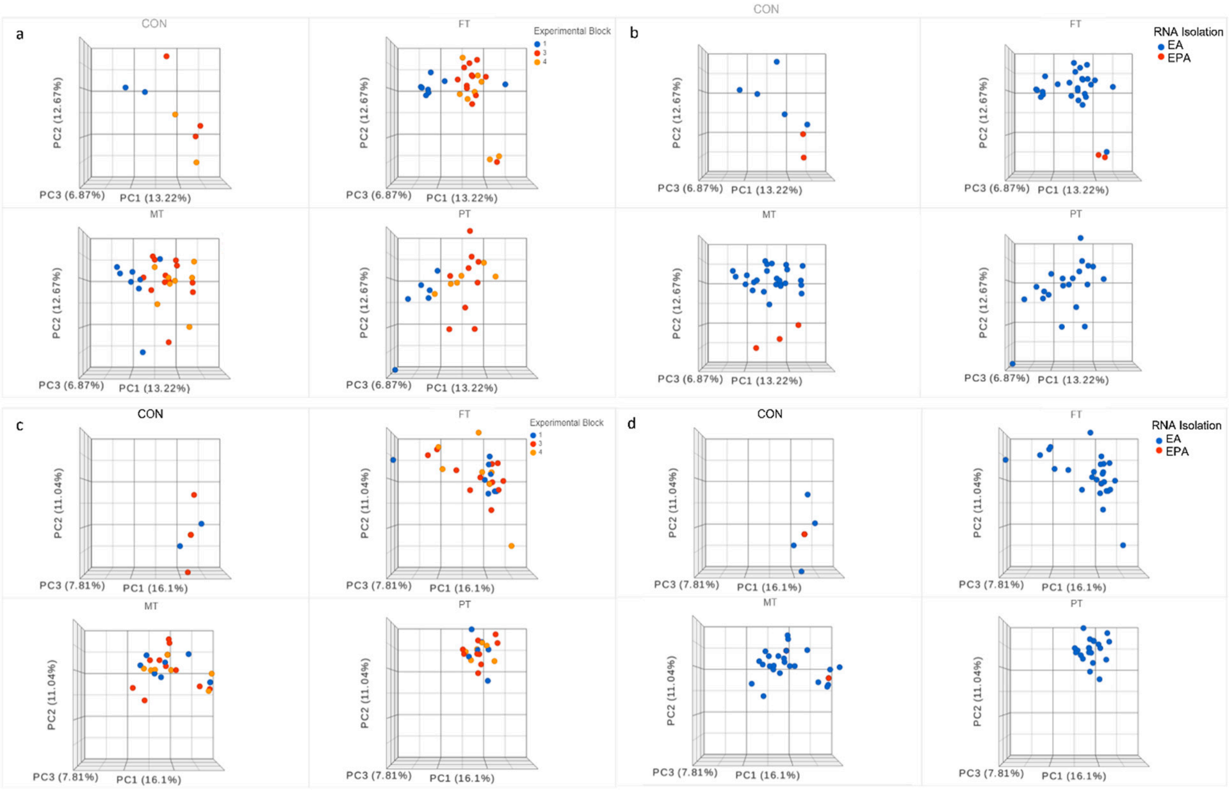

Figure S1. Principle component analysis of organophosphate pesticide gene counts from mouse liver study (a and b) before adjustment for batch effects relating to experimental blocking and RNA isolations, respectively and (c and d) after adjustment for batch effects resulting from experimental blocking and RNA isolations, respectively. Batch effects adjustment completed using a general linear model in Partek.

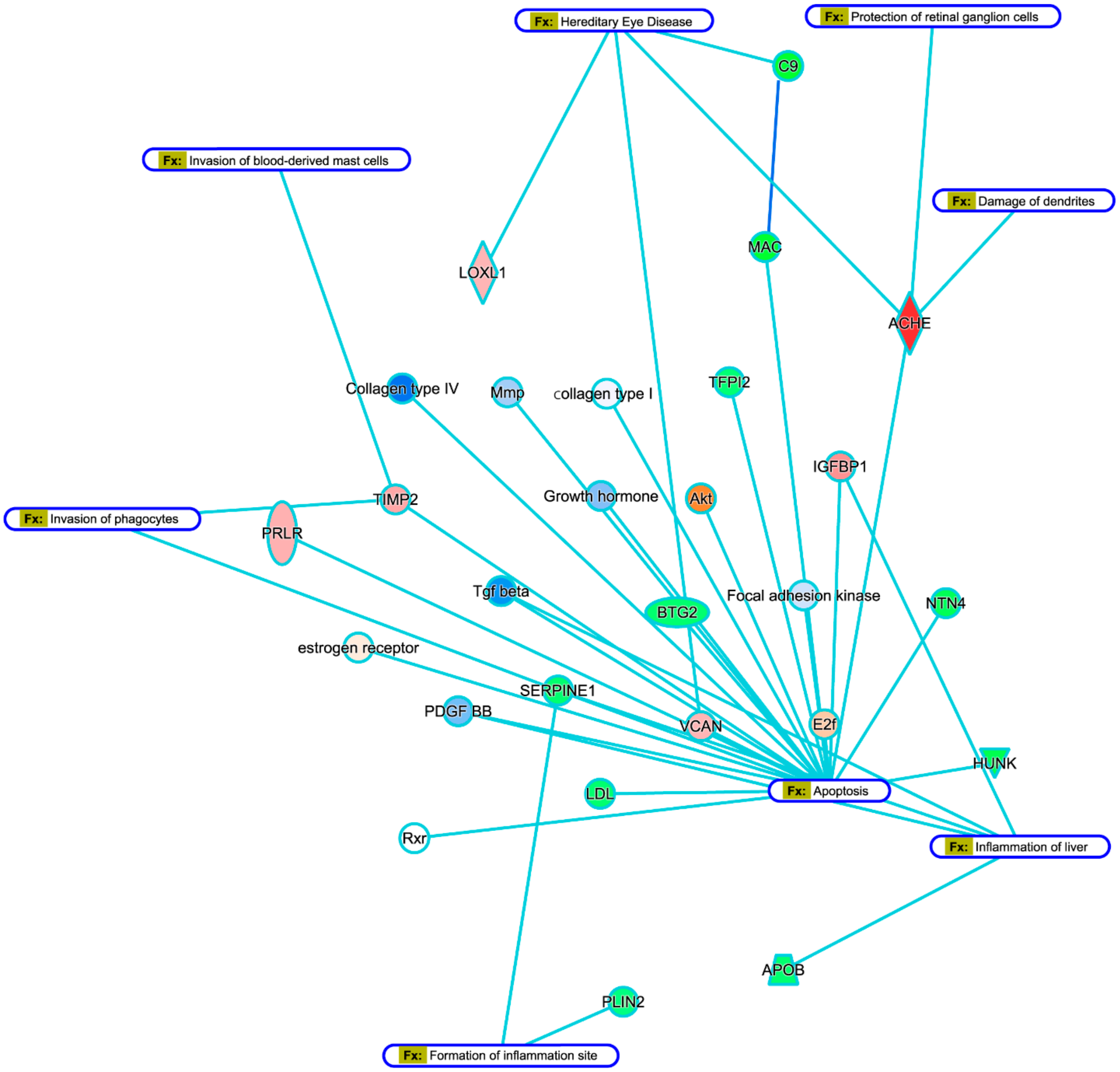

Figure S2. Impact of organophosphate pesticide exposure on diseases and functions (Interactive network ingenuity Pathway Analysis (IPA)).
